# Display of Native SARS-CoV-2 Spike on Mammalian Cells to Measure Antibody Affinity and ADCC

**DOI:** 10.21769/BioProtoc.5119

**Published:** 2024-11-20

**Authors:** Rebecca E. Wilen, Annalee W. Nguyen, Ahlam N. Qerqez, Jennifer A. Maynard

**Affiliations:** Department of Chemical Engineering, University of Texas at Austin, Austin, TX, USA

**Keywords:** SARS-CoV-2, Antibody affinity K_D_, Flow cytometry, Spike protein, Native spike variants, ADCC

## Abstract

*_The_* COVID-19 pandemic led to the rapid development of antibody-based therapeutics and vaccines targeting the SARS-CoV-2 spike protein. Several antibodies have been instrumental in protecting vulnerable populations, but their utility was limited by the emergence of spike variants with diminished susceptibility to antibody binding and neutralization. Moreover, these spike variants exhibited reduced neutralization by polyclonal antibodies in vaccinated individuals. Accordingly, the characterization of antibody binding to spike variants is critical to define antibody potency and understand the impact of amino acid changes. A key challenge in this effort is poor spike stability, with most current methods assessing antibody binding using individual domains instead of the intact spike or variants with stabilizing amino acid changes in the ectodomain (e.g., 2P or HexaPro). The use of non-native spike may not accurately predict antibody binding if changes lie within the epitope or alter epitope accessibility by altering spike dynamics. Here, we present methods to characterize antibody affinity for and activity against unmodified SARS-CoV-2 spike protein variants displayed on a mammalian cell membrane that recapitulates the native spike environment on infected cells. These include a flow cytometry–based method to determine the effective antibody binding affinity (KD) and an antibody-dependent cellular cytotoxicity (ADCC) assay to assess Fc-mediated activities. These methods can readily evaluate antibody activity across a panel of spike variants and contribute to our understanding of spike/antibody co-evolution.

Key features

• Allows rapid characterization of antibody binding to native SARS-CoV-2 spike on the mammalian cell surface.

• Describes analysis of antibody binding to multiple native spike variants without stabilizing mutations

• Describes analysis of Fc-mediated antibody-dependent cellular cytotoxicity

• Requires transient transfection of Expi293F and 293T cells to assess antibody binding and ADCC, a flow cytometer for antibody binding, and a plate reader for ADCC

• Protocol is readily adaptable to other viral fusogens and membrane proteins

## Graphical overview


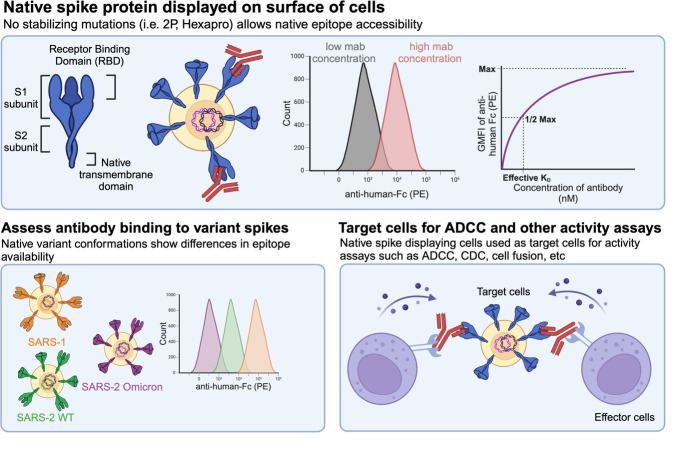


## Background

There is widespread interest in therapeutics to treat COVID-19 by targeting the spike protein of SARS-CoV-2 (hereafter referred to as “spike”). This type I viral fusogen mediates fusion between the virion and host cell membranes, necessary for infection, and is the primary target of the SARS-CoV-2 antibody response [1]. Between 2020 and 2023, six different spike-binding antibody therapeutics received emergency use authorization from the FDA but exhibited reduced activity against subsequent spike variants whose residue changes altered antibody binding and neutralization [2]. These variants were also less susceptible to neutralization by polyclonal antibodies elicited by vaccination with the original Wuhan-Hu-1 spike variant [3]. Antibody binding affinity and ability to recruit Fc-dependent immune responses against different spike variants can determine antibody potency, the biochemical impact of spike changes, and implications for protection.

However, analyses of antibody/spike interactions are complicated by the structure-function relationships of this large, homotrimeric protein. Each spike protomer comprises a conserved S2 stalk and a more variable receptor-binding S1 subunit. Host cell infection is initiated when the receptor binding domain (RBD) within S1 engages an ACE2 receptor on a host cell. Since the RBDs are primarily present in a “down” position, which shields the ACE2 binding site from immunological surveillance, this interaction requires an RBD to transiently sample the “up” state in order to be accessible for receptor binding [4]. The RBD/ACE2 interaction triggers a cascade of spike conformational changes: The S2’ cleavage site in the S2 subunit is exposed, allowing cell surface proteases (typically TMPRSS2 and cathepsin L) access to cleavage sites. Proteolysis releases the S1 subunit, freeing the hydrophobic fusion peptide to embed itself in the target cell membrane. The S2 domain then collapses from a meta-stable pre-fusion state into a thermodynamically more stable post-fusion conformation, resulting in the fusion of the viral and target cell membranes [5]. These structural rearrangements present challenges for the analysis of antibody/spike interactions as epitopes can be occluded or exposed depending on the immediate state of the spike protein.

Biochemical and immunological characterization of antibody/spike interactions is key to understanding spike antigenicity, elucidating mechanisms of protection and supporting the development of potent antibody therapeutics. However, the spike variant used, experimental conditions, and any changes to the spike sequence (e.g., stabilizing mutations, truncations to support soluble expression, or glycosylation differences) can impact the relevance of experimental measurements to human infection. Mutations throughout the spike protein can directly impact the ratio of spike found in “open” or “RBD-up” forms vs. “closed” or “RBD-down” forms, the ratio of pre- vs. post-fusion spike conformations, and antibody access to epitopes buried deeply in S2 [6–8]. In particular, low endosomal pH values (pH ~5–6) drive spike toward a more “closed” conformation, which can shield neutralizing epitopes [7,9]. Storage temperature can also influence spike stability—hydrogen-deuterium exchange mass spectrometry data indicate that incubation at 4 °C induces a reversible conformation shift toward an “open” state that exposes portions of the S2 interface [6,10]. The range of spike conformations sampled during evaluation can impact antibody binding and interpretation of mechanisms of antibody-mediated protection.

Unfortunately, the native spike protein is unstable when expressed solubly, resulting in very low yields of aggregation-prone protein and presenting challenges for biochemical analyses. The introduction of two stabilizing proline substitutions (K986P and V987P, also called the “2P” variant), was essential for determining the first cryo-EM structure of SARS-CoV-2 spike [11]. These 2P changes were subsequently included in multiple approved vaccines and vaccine candidates since they dramatically improve protein yield and storage stability [11,12]. A second-generation spike called HexaPro comprises the 2P changes plus four additional proline substitutions to further increase the yield and stability of the pre-fusion spike [13]. Additionally, most forms of soluble spike protein replace the native transmembrane region with a foldon domain, which may impact global protein dynamics [14]. While stabilized spike variants have been pivotal for enabling spike research, they also introduce non-native mutations. Since antibody binding is a result of both direct epitope–paratope interactions and indirect epitope accessibility, these stabilizing changes can impact effective antibody affinity by indirectly altering global spike dynamics as well as directly altering the epitope sequence. As a result, antibody binding results with stabilized spike variants may not accurately reflect interactions with native spike.

Strategies to characterize spike-binding antibodies have primarily used isolated spike subunits or stabilized spike variants. These approaches fuse the native RBD to the surface of yeast [15,16] or display truncated spike variants (such as the isolated RBD) or stabilized intact spike variants on mammalian cells [17,18], which allows researchers to measure antibody binding in a high throughput manner. However, these data reflect antibody binding to fully exposed RBDs, which may not predict binding to intact spike on the viral or infected cell surface. Other reports fused stabilized spike trimers to non-native transmembrane domains including the PDGFR homodimer, which may also impact antibody binding behavior [18]. Lentiviral pseudovirus assays using native spike on the surface of VSV-G-based pseudoviruses and ACE2 expressing 293T or Vero E6 target cells have been successfully used to evaluate antibodies [19–21]; however, these assays are complex and require increased safety precautions.

In addition to antibody binding, Fc-mediated effector functions are increasingly recognized for their contributions to protection. The antibody-dependent cellular cytotoxicity (ADCC) activity of anti-spike antibodies has been reported with similar methods that rely on different reporter or target cells than those used in this protocol [22,23]. Chen et al. used T-REx-293 cells stably expressing EGFP, luciferase, and codon-optimized native spike with the same NK-92 cell line used below. Hong et al. used Expi293F cells stably expressing red fluorescent protein and native spike with engineered Jurkat-luciferase NFAT-CD16 cells. While these protocols are effective in monitoring ADCC activity, they may require special user-created, engineered stable cell lines. As an alternative, this protocol demonstrates ADCC differences between anti-spike antibodies using two well-characterized human cell lines (293T and NK-92 cells) and transient transfection.

**Figure 1.**
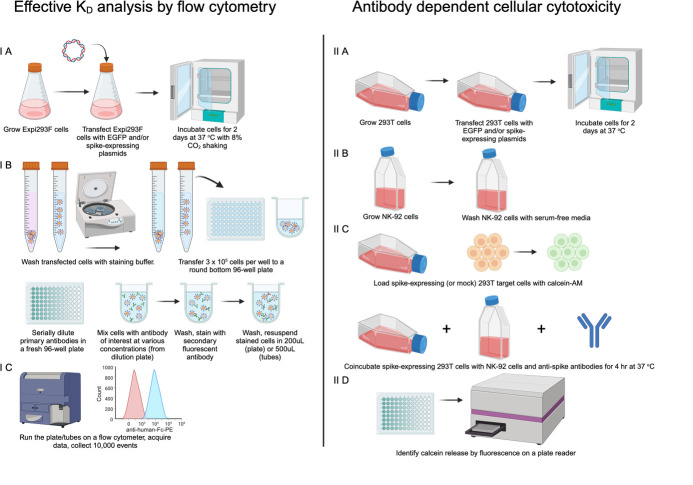
Overview of method. IA) Expi293F cells are transfected with EGFP- and spike-expression plasmids and incubated for two days at 37 °C. IB) Cells are washed and stained with anti-spike antibodies bearing human constant domains followed by detection with anti-human-Fc fluorescent antibody. IC) Cells are analyzed on a flow cytometer to assess anti-spike antibody binding by geometric mean fluorescence intensity (GMFI). IIA) 293T cells are grown, transfected with EGFP and spike-expressing plasmids, and incubated for 2 days at 37 °C. IIB) NK-92 cells are grown with recombinant human IL-2. IIC) Spike-expressing 293T cells are loaded with calcein-AM dye and then co-incubated with NK-92 cells and anti-spike antibodies at 37 °C. IID) After a 4 h co-incubation, the release of fluorescent calcein into the media from lysed target cells is measured using a plate reader. Created in BioRender. Wilen, R. (2024) BioRender.com/s02t921

We recently described antibodies that exhibit cross-reactive binding across SARS-CoV-1, SARS-CoV-2, and MERS-stabilized spikes [24]. The epitope recognized, spanning residues 985–1001 near the HR1/CH hinge in the pre-fusion spike S2 domain, is shielded in closed spike conformations and overlaps with the stabilizing “2P” changes. The antibodies analyzed include 3A3, which binds stabilized but not native SARS-CoV-2 Wuhan-Hu-1, SARS-CoV-1, and MERS spikes, and its engineered derivative RAY53, which binds both stabilized and native spike from SARS-CoV-2 Wuhan-Hu-1 and MERS and the stabilized form of SARS-CoV-1. Although both antibodies bind soluble, stabilized spike from these three β-coronaviruses with <10 nM affinity, the cryptic location of this epitope at the S2 apex overlapping the 2P residues suggests that the stabilizing changes alter antibody binding. To understand antibody interactions with this epitope in the context of native spike, we expressed spike with its native transmembrane domain and no stabilizing changes on the surface of Expi293F or 293T cells. In this protocol, native spike-expressing cells are used to measure the effective binding affinity, adapted from previously described protocols [25,26], and to assess susceptibility to Fc-mediated ADCC, using a cytotoxic assay that evaluates antibody ability to clear infected cells (Figure 1).

## Materials and reagents

**Biological materials**

1. Expi293F cells (Thermo Fisher Scientific, catalog number: A14527)
2. 293T cells (Millipore Sigma, catalog number: 12022001)
3. NK-92 cells expressing the high-affinity CD16a allele V158 (Brink Biologics, catalog number: haNK CD16-V158.NK-92.05; formerly, ATCC, catalog number: PTA-8836)

**Reagents**

1. Expi293F Transfection Kit (Thermo Fisher Scientific, catalog number: A14524). Includes ExpiFectamine 293 reagent
2. pHDM vector SARS-CoV-2 Wuhan-Hu-1 Spike glycoprotein (BEI Resources, catalog number: NR-52514); referred to here as pWT-SARS-2
3. pEGFP plasmid as described in Nguyen et al. [27]
4. Control anti-spike antibody S309 with human constant domains (constructed based on Pinto et al. [28]; also called Sotrovimab and commercially available from Thermo Fisher, catalog number: MA5-42316)
*Note: Select a control antibody binding a highly accessible epitope available both in the “open” and “closed” spike conformations, such as S309/Sotrovimab, which strongly binds SARS-CoV-1 and SARS-CoV-2 variants Wuhan-Hu-1 through Omicron XBB.1.5 and BQ.1.1. However, S309/Sotrovimab poorly binds spike variants starting after variants CH.1.1 and CA.3.1 [29].*
5. Anti-human-Fc-PE antibody or equivalent anti-human-Fc secondary antibody (Jackson ImmunoResearch, catalog number: C840K53)
6. Lipofectamine 3000 kit (LifeTech, catalog number: L3000008)
7. Recombinant human IL-2 (Millipore Sigma, catalog number: SRP3085-50UG)
8. Dulbecco’s phosphate buffered saline (PBS) (Millipore Sigma, catalog number: D8537)
9. Fetal bovine serum (FBS) (Gibco, catalog number: A5267-01)
10. Penicillin-Streptomycin (Millipore Sigma, catalog number: P4458)
11. Inositol (Millipore Sigma, catalog number: I7508)
12. 2-mercaptoethanol (Millipore Sigma, catalog number: M6250)
13. Folic acid (Millipore Sigma, catalog number: F8758)
14. Heat-inactivated horse serum (Millipore Sigma, catalog number: H1270)
15. Triton-X (Fisher Scientific, catalog number: BP151-100)
16. Sodium dodecyl sulfate (SDS) (Fisher Scientific, catalog number: BP166-500)
17. NaCl (Millipore Sigma, catalog number: S9888-10KG)
18. EDTA, disodium salt dihydrate (Fisher Scientific, catalog number: S311)
19. Trypan blue (MP Biomedicals, catalog number: 02195532-CF)
20. Expi293F expression media (Thermo Fisher Scientific, catalog number: A1435101)
21. OptiMEM (Gibco, catalog number: 31985070)
22. DMEM media (Millipore Sigma, catalog number: D5796)
23. Alpha minimum essential media (Thermo Fisher Scientific, catalog number: 12561056)
24. Expi293F Transfection Kit protocol (Thermo Fisher Scientific, catalog number: A14524, publication number: MAN0007814)

**Solutions**

1. Staining buffer (see Recipes)
2. 293T growth media (see Recipes)
3. Serum-free 293T growth media (see Recipes)
4. NK-92 growth media (see Recipes)
5. Lysis buffer (see Recipes)

**Recipes**

1. **Staining buffer**
  500 mL of PBS
  1% v/v FBS
2. **293T growth media**
  440 mL of DMEM
  10 mL of penicillin-streptomycin
  50 mL of FBS
3. **Serum-free 293T growth media**
  490 mL of DMEM
  10 mL of penicillin-streptomycin
4. **NK-92 growth media**
  500 mL of Alpha minimum essential medium without ribonucleosides and deoxyribonucleosides
  0.2 mM inositol
  0.1 mM 2-mercaptoethanol
  0.02 mM folic acid
  200 U/mL human IL-2 to start cultures, 100 U/mL for maintenance media
  12.5% heat-inactivated horse serum
  12.5% heat-inactivated FBS
5. **Lysis buffer**
  2% Triton-X
  1% SDS
  100 mM NaCl
  1 mM EDTA

**Laboratory supplies**

1. 96-well, U-bottom plate (Fisher Scientific, catalog number: 07-200-95)
*Note: Can be replaced by flow tubes or microcentrifuge tubes.*
2. 96-well, black plate with a flat, clear bottom (Fisher Scientific, catalog number: 07-200-625)
3. 1.5 mL microcentrifuge tubes (Fisher Scientific, catalog number: 05-408-129)
4. Pipette tips (1000, 200, and 10 μL) (Fisher Scientific, catalog number: 02-707-401, 02-708-416, 02-707-437)
5. Hemocytometer (Sigma-Aldrich, catalog number: Z375257)
6. 125 mL shaking flask for Expi293F (Fisher Scientific, catalog number: PBV12-5)
7. Micropipettes (Fisher Scientific, catalog number: F167380)

## Equipment

1. Biosafety cabinet (Thermo Scientific, catalog number: 1326122CON)
2. Incubator (37 °C, 8% CO_2_, shaking) for Expi293F (Infors HT, catalog number: I80002)
3. Incubator (37 °C, 5% CO_2_, stationary) for 293T and NK-92 (Eppendorf, catalog number: 6734010015)
4. Microscope (Olympus, catalog number: CKX41SF)
5. Tabletop centrifuge (Eppendorf, catalog number: 5405000441)
6. Vacuum trap system (BrandTech, catalog number: 20727403PM)
7. BD Fortessa LSR Cytometer or equivalent with at least two-color capability (BD Biosciences)
8. Microplate reader capable of reading fluorescence (excitation: 488, emission: 515) (Agilent BioTek, catalog number: SH1M-SN)

## Software and datasets

1. FlowJo or similar FSC analysis software (version 10.7.1)
2. GraphPad Prism or similar graphing/analysis software such as Microsoft Excel (version 9.5.0)

## Procedure

**Part I. Determine effective antibody K_D_**

- **Transfect Expi293F cells for spike display (day 0–1)**
  Stabilization of the membrane-bound spike protein enhances overall stability and expression but also alters the spike conformational dynamics. Here, we describe the expression of native spike protein on the mammalian cell surface, which better mimics the dynamics of native protein on SARS-CoV-2-infected cells. Expi293F cells are transiently transfected with a plasmid encoding the entire spike ectodomain and its native transmembrane domain. Although the plasmid was originally designed for use in packaging lentivirus with SARS-CoV-2 spike on the viral surface, it works well for direct transient expression in mammalian cells. Because liposomal transfection results in multiple plasmids entering each cell, co-transfection with an EGFP-expressing plasmid is used to identify successfully transfected cells. After two days of expression, cells can be stained with anti-spike antibodies and fluorescent secondary antibodies to analyze antibody binding (section B).
*Note: This protocol is adapted from the Expi293F Transfection Kit protocol.*
  1. Thaw and grow Expi293F cells for at least one week after thawing.
  2. Count cells and determine viability.
    - Dilute a sample of cells 10-fold with PBS (i.e., 100 μL cells + 900 μL PBS).
      Undiluted cells should be approximately 3–5 × 10^6^ cells/mL so diluted cells will be approximately 3–5 × 10^5^ cells/mL, a more manageable amount to manually count.
    - Mix 10 μL of diluted cells with 10 μL trypan blue.
    - Use a hemocytometer and inverted microscope or automated cell counter to determine cell count and viability.
*Note: Cell viability should be >95%. If viability is lower, re-seed cells for maintenance and proceed at a later date.*
  3. Determine the number of cells required for the experiment and seed 1.5× that number at 2.5 × 10^6^ cells/mL.
    - We recommend staining 3 × 10^5^ cells/sample in microcentrifuge tubes or per well of a 96-well plate.
    - Example: 3 × 10^5^ cells/sample × (6 concentrations + 3 controls) × 2 replicates = 5.4 × 10^6^ cells needed × 1.5 overage = at least 8.1 × 10^6^ mock-transfected and spike-transfected cells to seed at 2.5 × 10^6^ cells/mL.
    - We recommend transfecting 1.5× the number of cells needed for the experiment to account for excess; however, the exact number transfected may need to be adjusted to account for available tissue culture plates.
      Example: 18 samples require 3.25 mL of cells at 2.5 × 10^6^ cells/mL, which can be rounded up to 4 mL to fit available tissue culture plates (i.e., 2 wells with 2 mL each in a 6-well plate)
  4. Incubate cells at 37 °C and 8% CO_2_ with shaking at 125 rpm.
  5. Next day, repeat step A2 to determine cell count and viability.
*Note: Viability should be >95%. If cell viability is less than 95%, do not proceed and repeat steps A1–4.*
  6. Dilute cells to 2.5 × 10^6^ cells/mL and seed the appropriate number of cells calculated in step A3.
    Example: 18 samples calculated above require approximately 4 mL of culture at 2.5 × 10^6^ cells/mL.
  7. Calculate the amount of DNA and other reagents necessary for the experiment.
    - pEGFP plasmid: 0.5 μg/mL of culture to transfect.
    - pWT-SARS-2 plasmid: 0.5 μg/mL of culture to transfect.
    - OptiMEM: 60 μL/mL of culture to transfect.
      Fresh OptiMEM is needed to dilute both the DNA and ExpiFectamine, so two aliquots of OptiMEM should be prepared per transfection.
    - ExpiFectamine 293: 3.2 μL/mL of culture to transfect.
    - Example: For 4 mL of cell culture to be transfected, use 2 μg of pEGFP plasmid, 2 μg of pWT-SARS-2 plasmid, 240 μL of OptiMEM for DNA dilution, 240 μL of OptiMEM for ExpiFectamine dilution, 12.8 μL of ExpiFectamine 293.
  8. Combine pEGFP plasmid and pWT-SARS-2 plasmid in OptiMEM. Use the EGFP plasmid only for a mock transfection control lacking spike expression to evaluate antibody specificity.
    - Mix by gently pipetting up and down, inverting the tube, or swirling.
*Note: DNA should be at a concentration* ≥ *1 μg/mL, treated with an endotoxin removal column to remove residual endotoxin from plasmid purification, and sterile-filtered (0.2 μm) to prevent contamination of the Expi293F cultures.*
  9. Dilute ExpiFectamine 293 reagent in fresh OptiMEM.
    - Mix by gently pipetting up and down.
    - Incubate at room temperature for 5 min.
*Note: The ExpiFectamine dilution can be pooled into a single tube at this step and aliquoted to each transfection (mock, SARS-CoV-2 spike, other spike variants) in the next step.*
  10. Add the diluted ExpiFectamine (step A10) to the diluted DNA (step A9) and mix gently by pipetting, swirling, or inverting the tube. Incubate the mixture for 10–20 min at room temperature.
  11. Add DNA–ExpiFectamine 293 mixture dropwise to the diluted cells from step A6 and gently swirl to mix.
  12. Incubate cells in an incubator at 37 °C and 8% CO_2_ with shaking at 125 rpm for 48 h.
*Note: Expi293F transfection typically includes adding enhancers to the cell culture the day following transfection. This addition is optional. We have not noticed that the addition of enhancers results in a difference in surface spike expression levels two days after transfection.*
- **Flow cytometry staining (day 3)**
  The Expi293F cells expressing native spike protein (Step A) can be used to characterize anti-spike antibodies. Anti-spike antibodies are serially diluted and incubated with spike-expressing Expi293F cells. These are then washed, incubated with a secondary detection antibody, washed again, and analyzed by flow cytometry as described in section C. We recommend several key controls including (i) no primary antibody to assess non-specific binding by the secondary antibody used (hereafter referred to as “secondary only”), (ii) a negative isotype control antibody with the same constant domains as the test antibody but different variable regions such that it does not bind spike, and (iii) a positive control antibody that is known to bind spike. It is important to choose a positive control antibody (such as S309) that binds an epitope available on various coronaviruses as well as an epitope that is on a portion of the SARS-CoV-2 spike accessible in various conformations (“up” vs. “down” vs. “open”).
  The Langmuir isotherm analysis used to measure the effective K_D_ requires that the soluble binding partner (here, antibody) always be present in excess. This allows the experiment to comply with the model assumption that the free antibody concentration remains constant throughout the experiment, regardless of the amount of antibody/ligand complex formed. At low antibody concentrations, this may require the use of large volumes to provide a greater number of antibodies than spike proteins. Appropriate volumes can be determined from a simple experiment: Stain an equal number of cells with a single, low antibody concentration in different final volumes (e.g., 10 nM antibody in 50 μL, 100 μL, 500 μL, 1 mL, and 5 mL) before staining with secondary antibody and flow cytometric analysis. If the number of antibody molecules limits antibody/spike complex formation, the GMFI of the stained cells will increase with increasing volume. The smallest volume resulting in maximal observed GMFI should be used for affinity measurements.
  The protocol below references the example plate layout in Figure 2. Briefly, antibody dilutions are prepared in the preparative Plate A at 2× final concentration [sufficient volume for at least 4 wells (>100 μL): two replicates each of two cell types at 25 μL of antibody solution per well]. In the assay Plate B, 25 μL of cells are aliquoted into each well, and then 25 μL of serially diluted antibody from Plate A is added for a 1:1 dilution to the final cell and antibody concentrations. Typical final cell concentration is 6 × 10^6 ^mL at 50 μL per well, while the antibody range will depend on antibody affinity; however, 300 nM to ~1 nM is recommended for the example antibody used here, RAY53.
  1. On day 2 post-transfection, count the transfected or mock-transfected cells using a hemocytometer or automated cell counter.
  2. Determine the number of cells needed for the experiment and transfer 1.5× the calculated number to a 15 mL conical tube.
    - We recommend staining 3 × 10^5^ cells per sample (50 μL of final volume at 6 × 10^6^ cells/mL final concentration) in a 96-well plate; however, this may need to be optimized depending on the flow cytometer used.
    - Example: 3 × 10^5^ cells per sample × (6 concentrations + 3 controls) = 5.4 × 10^6^ cells needed × 1.5 overage = at least 8.1 × 10^6^ cells transferred to a conical tube.
  3. Wash twice with staining buffer.
    - Centrifuge at 250× *g* for 5 min.
    - Aspirate media.
    - Resuspend cells in at least ~600 μL of staining buffer per 10^6^ cells.
      Example: 8.3 × 10^6^ cells should be resuspended in at least ~5,000 μL of staining buffer.
    - Repeat step B3a.
*Note: Washing can lead to cell losses. The recommended resuspension volume should lead to a cell concentration of ~1.6 × 10^7^ cells/mL with no cell losses, which is above the final concentration required, allowing the user to dilute the cells to their final concentration and reducing the need for additional spins to prepare the cells.*
  4. Count cells and dilute to 1.2 × 10^7^ cells/mL in staining buffer.
    - Dilute cells 20-fold (i.e., 5 μL of cells + 95 μL of PBS) to ~8 × 10^5^ cells/mL.
    - Mix 10 μL of diluted cells with 10 μL of trypan blue.
    - Use a hemocytometer and inverted microscope or automated cell counter to determine cell count and viability.
  5. Dilute test antibody and controls in a fresh dilution plate (Figure 2A, Plate A).
    - Prepare enough antibody solution for at least two replicates at 25 μL per replicate plus 20 μL excess.
*Note: Multiply the number of replicates by the number of cell types tested. For example, two replicates and two cell types should be treated as four replicates worth of antibody needed. The following protocol describes two replicates and two cell types (four replicates worth of antibody) for each antibody concentration.*
      For four replicates: Start with 120 μL of staining buffer per well (25 μL × 4 replicates + 20 μL excess = 120 μL). For a 5-fold serial dilution, 30 μL will be added, mixed, and transferred to the subsequent dilution.
    - Prepare the highest antibody concentration at 600 nM (2× the final desired highest concentration) and serially dilute 5-fold at least six times.
      i. Add 120 μL of staining buffer to wells A2–A7 of Plate A (repeat in additional row per antibody testing).
      In the example in Figure 2A, Antibody 1 is diluted in row A. Antibody 2 is diluted in row C, so 120 μL of staining buffer was also added to wells C2–C7.
      ii. Add 150 μL of 600 nM antibody to well A1.
      In the example in Figure 2A, 150 μL of 600 nM Antibody 2 should be added to well C1.
      iii. Transfer 30 μL from A1 to A2 and mix thoroughly >6 times.
      In the example in Figure 2A, 30 μL from C1 should also be transferred to C2 and mixed thoroughly.
      iv. Repeat, transferring 30 μL of A2 to A3 and mixing thoroughly, 30 μL of A3 to A4, etc., until A7.
      In the example in Figure 2A, this should be repeated by serially diluting from C2 to C7.
*Note: Assay controls include:*
*i. Positive control antibody to confirm spike display: Samples stained with S309 or other positive control antibody to detect spike on the cell surface (25 μL per replicate well at 10 nM).*
*ii. Secondary-only control: Sample not stained with an anti-spike antibody (no antibody control/secondary only control) (25 μL of staining buffer).*
*iii. Cells-only control: Sample lacking both anti-spike primary antibody and secondary (25 μL of staining buffer).*
*Note: Concentrations used may need to be optimized for different antibodies. We recommend initial tests starting 10–50× above the expected K_D_. If the affinity is unknown, we recommend starting at 300 nM final concentration. The dilution factor can also be adjusted for each antibody to encompass the full curve.*
  6. Aliquot 25 μL of diluted cells (3 × 10^5^ total cells) per sample in a fresh 96-well plate (Figure 2B, Plate B).
  7. Transfer 25 μL of stain from the stain dilution plate (step B5, Plate A) to appropriate wells of cells (step B6, Plate B), mix gently by pipetting, and incubate for 1 h on ice. In this example, the stain prepared in wells A1–A9 in plate A is added to wells A1–A9, B1–B9, E1–E9, and F1–F9 in plate B. The stain prepared in wells C1–C8 in plate A is added to wells C1–C8, D1–D8, G1–G8, and H1–H8 in plate B.
  8. Wash twice with staining buffer.
    - Centrifuge at 250× *g* for 5 min.
    - Aspirate media.
    - Resuspend cells in 200 μL of staining buffer.
    - Repeat steps B8a and B8b.
  9. Incubate cells with 50 μL of 1:250 goat anti-human-Fc-PE in staining buffer for 1 h on ice.
*Note:*
    - *If using a different secondary antibody, the dilution factor may need to be optimized.*
    - *For cells-only control, it is appropriate to replace the secondary stain with 50 μL of staining buffer.*
  10. Repeat step B8 with the final resuspension in 200 μL of staining buffer or the volume required for analysis on the flow cytometer used.
    **Figure 2.**
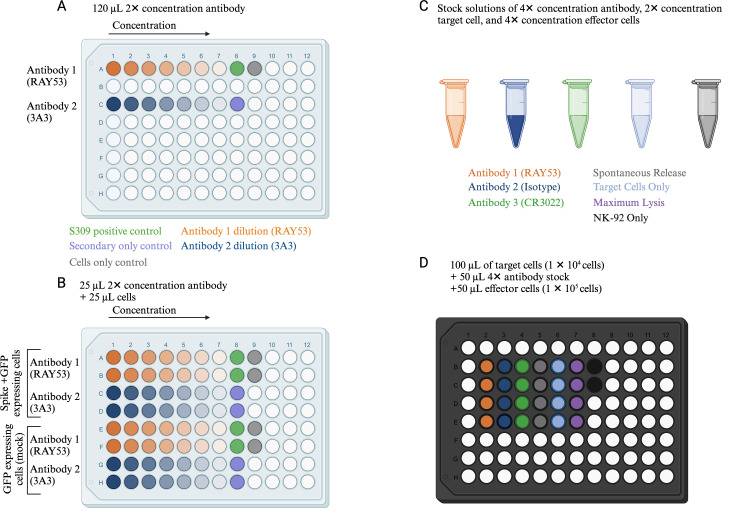
Example plate layouts for antibody staining for Part I, section B (antibody K_D_, a–b) and Part II, section C (ADCC, c–d). A. The antibody is diluted in a fresh plate (Plate A) to create 2× concentrated master mixes with enough volume for all necessary replicates (25 μL per replicate plus excess to allow for pipetting). B. 3 × 10^5^ total cells (25 μL, 1.2 × 10^7^ cells/mL) are mixed with 25 μL of antibody master mix from Plate A in the staining plate (Plate B). C. Cells and antibodies for antibody-dependent cellular cytotoxicity (ADCC) are diluted in tubes to create concentrated master mixes with enough volume for all necessary replicates (4× concentrated for antibodies, 1 × 10^5^ cells/mL for target cells, 2 × 10^6^ cells/mL for effector cells). D. 1 × 10^4^ target cells (100 μL, 1 × 10^5^ cells/mL), 1 × 10^5^ NK-92 cells (50 μL, 2 × 10^6^ cells/mL), and 4× concentrated antibody (50 μL) are combined. Created in BioRender. Wilen, R. (2024) BioRender.com/c65x019
*Note: Plates or tubes can be used in both panel A and panel C. Tubes may be preferred for simplicity; however, as the number of antibody samples increases, a plate allows for better organization.*
- **Flow cytometry data acquisition (day 3)**
  Expi293F cells expressing native spike and stained with anti-spike antibodies are analyzed by multi-color flow cytometry. Cells are first gated by size (FSC vs. SSC), then for singlets (FSC-A vs. FSC-H), and finally for EGFP-positive cells to identify the transfected population. These EGFP-expressing cells are then assessed for antibody binding, which is monitored by fluorescence from the secondary antibody. Key controls include secondary antibody only (negative control), an isotype control antibody (negative control), and a cross-reactive anti-spike antibody (positive control) in addition to the antibody of interest. Confocal imaging confirms GFP expression and S309 binding to Wuhan-Hu-1 spike on the cell surface (Supplemental Figure 1).
  1. Collect data on a flow cytometer.
    - Gating strategy:
      i. Cells: FSC A vs. SSC A, gate main cell population.
      ii. Singlets: FSC A vs. FSC H, gate only cells on the main diagonal; cells off the diagonal may be doublets or larger cell clumps.
      iii. EGFP+ cells: EGFP vs. FSC A, gate only EGFP+ cells.
    - Collect data for 10,000 EGFP+ cells.
*Note: Depending on individual instrument and filter settings, compensation may be necessary. Adjust voltages so that negative and positive samples appear on the screen and are not cut off by the cytometry software. Make sure to acquire EGFP and PE (or appropriate secondary fluorophore) fluorescence in addition to FSC and SSC channels.*
    - Example gating strategy is shown in Figure 3.
      Export either geometric mean fluorescence intensity (GMFI, used here) or median fluorescence intensity (MFI) for analysis to account for a non-normal distribution of points.
  2. Proceed to the data analysis section for details on analysis to determine the effective K_D_.
    **Figure 3.**
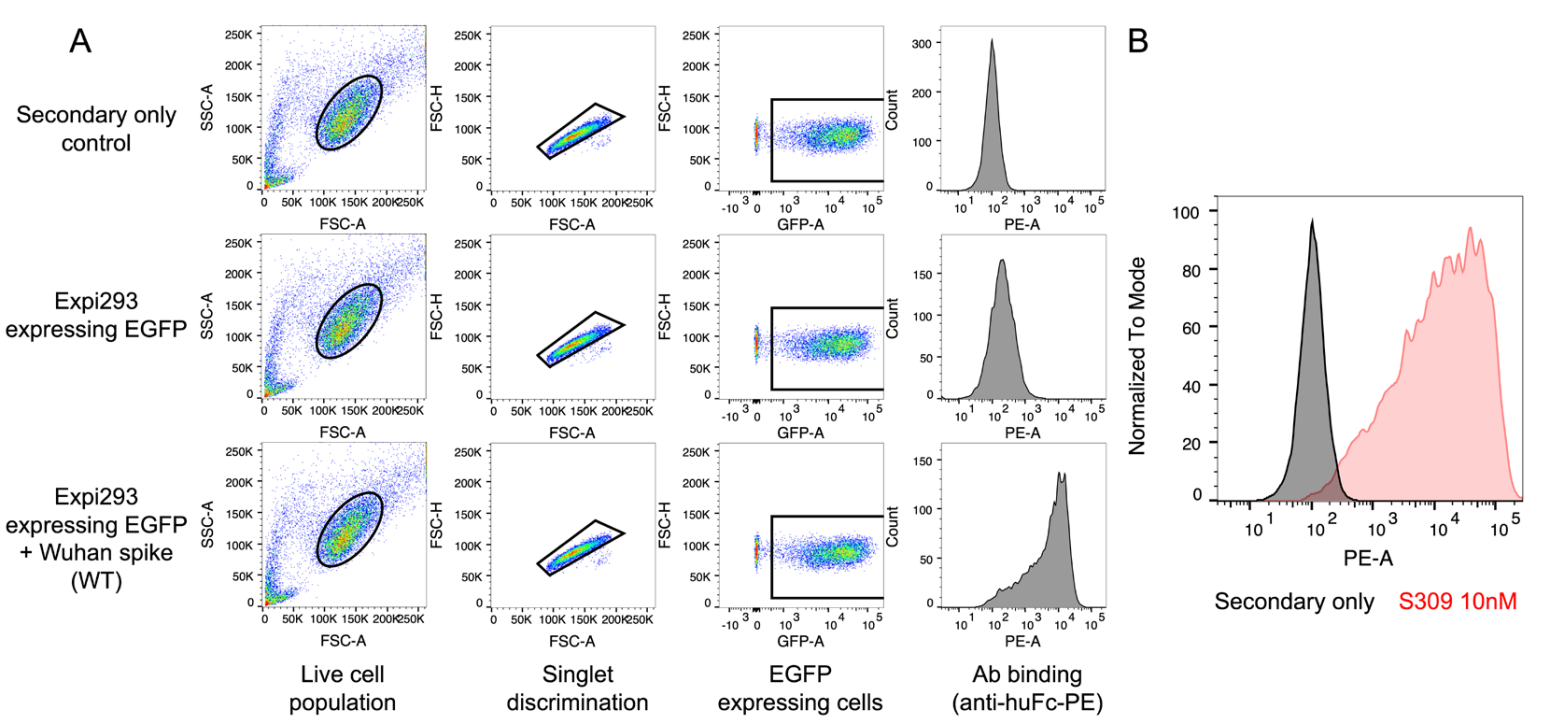
Gating strategy to isolate spike-displaying cells. Example results and gating strategy to determine the effective K_D_. A) Example gating for secondary-only control (top), RAY53 staining of Expi293F cells only expressing EGFP (middle), and RAY53 staining of Expi293F cells expressing both EGFP and spike (bottom). The sample is first gated for cell size (FSC vs. SSC), singlets (FSC-A vs. FSC-H), and EGFP expression; then, the PE fluorescence (Ab binding) of the final gated population is analyzed. The reported PE GMFI over several concentrations can be used to directly determine the effective antibody K_D_. B) Example shown of positive and negative control histogram after gating. Negative control (secondary only) in black, and positive control (S309 antibody) in red.

**Part II: Antibody-dependent cellular cytotoxicity**

- **Transfection of 293T cells with spike and EGFP plasmids (day 0–1)**
  In a similar protocol to Part I, section A above (Expi293F transfection), adherent 293T cells are transfected with both a plasmid for native spike protein expression and a plasmid for EGFP expression using Lipofectamine 3000. 293T and Expi293F cells are variants of HEK 293 cells that have different levels of protein expression, and the 293T cell variant used for both the following and previous protocols may need to be optimized. Cell media is replaced the day after transfection to remove transfection reagents. Passage the 293T cells before they reach confluency, as these cells can easily form clumps that are difficult to break up into single cells. As in Part I, we recommend including a “mock” transfection control lacking DNA to assess the specificity of the ADCC response.
  1. Passage 293T cells in 293T growth media as described [30].
*Note: It is important not to let the cells overgrow (>90% confluency), as this can cause clumps that can cause high error in this assay.*
  2. Seed cells such that there is a single flask prepared on the day of transfection at 70%–90% confluency for each spike variant or control tested.
    - Example: To assess ADCC with cells expressing only SARS-CoV-2 Wuhan-Hu-1 spike, two flasks should be prepared—one for transfecting with pWT-SARS-2 plasmid and one for mock transfection as a negative control.
    - Calculate the number of target cells required for each spike variant and scale the transfection to at least five times that number to ensure sufficient cell numbers. The 293T cell line typically reaches 2–3 × 10^5^/cm^2^ at 100% confluence. For each spike variant or control tested, triplicate wells containing 10^4^ transfected 293T per well for each control (target only, spontaneous release, maximum lysis) and each antibody concentration will be required.
    - Example: To assess ADCC of SARS-CoV-2 Wuhan-Hu-1 spike transfected cells incubated with 3A3 or RAY53 at a single concentration, 15 wells of spike-expressing 293T cells will be required (three replicates each of 3A3 experimental well, RAY53 experimental well, target-only control, spontaneous release control, and maximum lysis control) and a minimum of 7.5 × 10^5^ cells must be transfected. Although transfection of 70% confluent 293T cells in a single well of a 24-well plate (surface area ~2 cm^2^) would be sufficient, the recommended minimum transfection scale is a single well of a 6-well plate (~10 cm^2^) or a T25 flask (25 cm^2^) for ease of handling. We describe the transfection of a T25 flask of 293T cells here.
    - For a T25 flask with 4 mL of culture volume, seed 5 × 10^5^ cells per milliliter of 293T cells in 4 mL of 293T growth media the day before transfection.
  3. Dilute 1 μg of DNA per milliliter of culture volume and 2 μL of P3000 reagent per milliliter of culture volume into 50 μL of OptiMEM per milliliter of culture volume.
    For a T25 flask with 4 mL of culture volume, combine 4 μg of DNA, 8 μL of P3000 reagent, and 200 μL of OptiMEM in a microcentrifuge tube.
*Note: DNA stock should be at a concentration ≥ 1 μg/mL, treated with an endotoxin removal column to remove residual endotoxin from plasmid purification, and sterile-filtered (0.2 μm) to prevent contamination of the 293T cultures.*
  4. Dilute 2.5 μL of Lipofectamine 3000 reagent per milliliter of culture volume into 50 μL of OptiMEM per milliliter of culture volume.
    For a T25 flask with 4 mL of culture volume, combine 10 μL of Lipofectamine 3000 and 200 μL of OptiMEM in a microcentrifuge tube.
  5. Gently pipette diluted DNA/P3000 mixture into the tube containing diluted Lipofectamine 3000, mix gently by pipetting, swirling, or inverting the tube, and let sit for 10–15 min.
  6. Slowly add the DNA/Lipofectamine 3000 mixture in a dropwise manner to the prepared flask of 293T cells.
  7. Incubate at 37 °C overnight.
  8. The next day, remove media and replace with fresh 293T growth medium.
- **Preparation of NK-92 cells (day 1–2)**
  Approximately 90% of human NK cells express the classical Fc receptor CD16A, which is activated by clustered antibodies on target cells to initiate ADCC activities, but initial attempts at immortalizing NK-92 cells resulted in loss of CD16A expression [31,32]. Accordingly, NK-92 cell lines engineered to stably express the high (V158) or low (F158) alleles of CD16A are a popular option to assess ADCC in vitro [31,33,34]. These lines require human IL-2 to support growth in vitro and constitutively express EGFP. Before seeding into the assay plates, cells are washed to remove residual IL-2 and bovine IgG from the media. Bovine IgG may bind to CD16 on the NK cells, reducing available receptors and limiting the final ADCC response.
  1. Passage NK-92 cells using NK growth media described above, ensuring that sufficient healthy NK-92 cells will be available for the planned assay. Each well planned for the experiment will require 10^4^ NK-92 cells. Plan to have at least three times the number required available on the day of the assay. Achieving adequate numbers of NK-92 cells can be a limiting factor in this experiment, so incorporating the NK-92 growth timeline in the experimental plan is essential.
    - Supplement media with 500 U human IL-2/mL initially upon thaw, followed by 250 U human IL-2/mL for subsequent passages.
      Note: IL-2 is sensitive to freeze-thaw and should be aliquoted into small aliquots after reconstitution. Once thawed, the IL-2 aliquots can be stored at 4 °C for up to one week.
    - Grow NK-92 cells with the T-flask placed vertically.
    - During passaging, cell densities should range between ~8 × 10^4^ cells/mL and ~3 × 10^5^ cells/mL.
    - Cells may be clumpy during passaging. Pipetting up and down with a serological pipette of appropriate volume may gently disperse clumps as cells trapped in clumps will be poorly responsive in the assay.
  2. One day after transfecting the 293T cells (day 1), centrifuge the NK-92 cells (250× *g* for 5 min) and aspirate the media.
  3. Replace with fresh NK-92 media, add fresh IL-2 (250 U human IL-2/mL), and incubate overnight at 37 °C.
  4. On the day of the assay (day 2), count the cells (Part I, section A2) and resuspend them in NK-92 growth media without FBS to a concentration of 2 × 10^6^ cells/mL.
*Note: Viability should be >95%. If cell viability is <95%, do not proceed and repeat Part II, section B1–3.*
- **Setup of ADCC (day 2)**
  To measure ADCC in terms of target cell lysis, spike-expressing 293T target cells are loaded with acetoxymethyl ester calcein (calcein-AM) dye. This hydrophobic dye diffuses across the cell membrane where it is cleaved by intracellular esterases to its fluorescent calcein form. Upon lysis, cells release fluorescent calcein into the media. After removing the cells and debris, media are transferred to a new plate and the fluorescence is measured by a plate reader. When preparing the spike-expressing 293T target cells, gently pipette to dissociate cell clumps, which can reduce calcein-AM loading into cells and subsequent release by ADCC. The procedure described below uses a 10:1 effector:target cell ratio; however, this can be optimized as needed. While edge effects from evaporation are unlikely during the short incubation, common practice is to avoid using the outer wells of the plate (column 1, column 12, row A, and row H) and fill these wells with PBS or excess media. Key controls include (i) a spontaneous release control with target cells, NK-92 cells, and no antibody present, (ii) an NK-92-only control, (iii) a target cell–only control, and (iv) a maximum lysis control with target cells only and lysis buffer added ~10–15 min before reading the plate absorbance.
  1. On day 2, remove media from 293T cells and wash with one culture volume of PBS.
    - For a T25, add 4 mL; for a T75, use 8–12 mL.
    - Repeat 2–3 times to remove non-adherent and dead cells.
  2. Remove PBS, add one culture volume of PBS with 10 mM EDTA, and incubate for approximately 10 min or until cells detach from the flask surface. Gentle tapping on the side of the flask can help release the cells from the surface of the flask.
*Note: Use EDTA rather than trypsin, as trypsin can cleave the spike proteins from the cell surface.*
  3. When cells are released from the plate (confirm by looking at the flask under an inverted microscope), add 2–3 mL of complete 293T growth media per milliliter of original culture and transfer to a 15 mL conical tube.
  4. Spin cells at 250× *g* for 5 min, remove supernatant, and resuspend in serum-free 293T growth media.
    Repeat steps IIC2–3 times to remove excess dead cells. Resuspend in serum-free 293T growth media after the final wash.
*Note: Use enough media to resuspend the cells at ~1 × 10^6^ cells/mL based on confluency and flask size. For example, one well of a 6-well plate at ~80% confluency should have approximately 1.6–2.4 × 10^6 ^cells, so the pellet should be resuspended in ~2 mL of 293T growth media.*
  5. Pipette cells up and down vigorously ~10 times with a 1 mL Pipetman or similar with a small opening to disrupt cell clumps.
*Note: The presence of cell clumps causes several issues for this assay. First, they will result in incorrect cell counting and inaccurate E:T ratios. Second, calcein-AM staining may be inconsistent across cell clumps. Third, clumpy cells will not properly engage target cells. Together, these issues impact assay reproducibility.*
  6. Count cells, determine viability, and dilute in serum-free 293T growth media to a concentration of 0.5–1 × 10^6^ cells/mL in a conical tube.
*Note: If a large fraction of dead cells appears at this stage (greater than 10%), the likelihood of a successful assay is low; repeat or optimize steps IIC1–6.*
  7. Add calcein-AM to the transfected cells to a final concentration of 2 μM, invert the tube several times to mix, wrap in foil, and incubate at 37 °C for 30 min, inverting every ~10 min.
  8. Wash transfected 293T cells three times with at least double the original culture volume of serum-free 293T growth media. For a T25, wash with at least 8 mL of complete serum-free 293T growth media.
*Note: The cell pellet will appear green due to calcein.*
  9. Resuspend transfected 293T cells in 1 mL of serum-free 293T growth media, count, and dilute cells to 1×10^5^ cells/mL.
    If cells are less than 1 × 10^5^ cells/mL, spin cells one more time and resuspend in appropriate volume.
  10. Prepare 4× final concentration antibody stock in microcentrifuge tubes (**Figure 2C**).
    For example, if a final concentration of 50 nM is required, the stock should be diluted to 200 nM.
  11. To each well in a 96-well plate (Figure 2D, Plate D):
    - Add 100 μL of transfected 293T cells (at 1 × 10^5^ cells/mL, 1 × 10^4^ cells/well).
      Use 100 μL of media only for the NK-92 only control wells.
    - Add 50 μL of antibody from the dilution plate to the assay plate.
      Replace with media for spontaneous release control, but do not add media or antibody to max lysis control.
    - Add 50 μL of NK-92 cells (at 2 × 10^6^ cells/mL, 1 × 10^5^ cells/well for an E:T ratio of 10:1).
      Do not add to max lysis control.
    - Max lysis control: 100 μL of transfected 293T cells only (1 × 10^4^ cells/well). These wells will have half the volume of the other wells during this incubation step.
    - NK-92-only control: 50 μL of NK-92 cells (1 × 10^5^ cells/well) + 150 μL of serum-free 293T growth media.
    - Spontaneous lysis control: 100 μL of transfected 293T cells (1× 10^4^ cells/well) + 50 μL of NK-92 cells (1 × 10^5^ cells/well) + 50 μL of serum-free 293T growth media.
    - In the final 200 μL of assay volume, antibody concentrations will be 50 nM, with an E:T ratio of 10:1 and 1.1 × 10^5^ total cells.
  12. Incubate plate (Plate D) at 37 °C for 4 h covered in foil.
- **ADCC readout and analysis (day 2)**
  After 4 h, the cells are centrifuged, and the media is transferred to a black plate. Calcein released into the media is detected via fluorescence on a plate reader.
  1. Remove the plate from the incubator and carefully add 100 μL of lysis buffer to the maximum lysis control wells to normalize its volume to that of experimental wells. Any contamination of other wells with the lysis buffer will impact the results of the assay. Allow the plate to sit for 5–10 min at room temperature in the dark.
  2. Spin the plate at 400× *g* for 10 min and transfer 100 μL of supernatant to a fresh black clear-bottom 96-well plate.
  3. Using a plate reader, measure the fluorescence intensity at an excitation wavelength of 488 nm and emission wavelength of 515 nm.

## Data analysis

**Part I:**

Upload your FSC files into FlowJo or an equivalent flow cytometry analysis software. Follow the gating scheme described above (Figure 3; Part 1, section C) to isolate the EGFP-expressing cell population. First, check for spike expression by analyzing positive and negative controls (S309 and secondary-only, respectively). Confirm controls are as expected (no shift for negative control and large shift for positive control) (Figure 3B); if not, do not proceed. An exception is in the case of a control known not to bind a particular spike variant; for example, antibody S309 can bind several SARS lineage β-coronavirus spikes but is known not to bind to the MERS spike.

**Figure 4.**
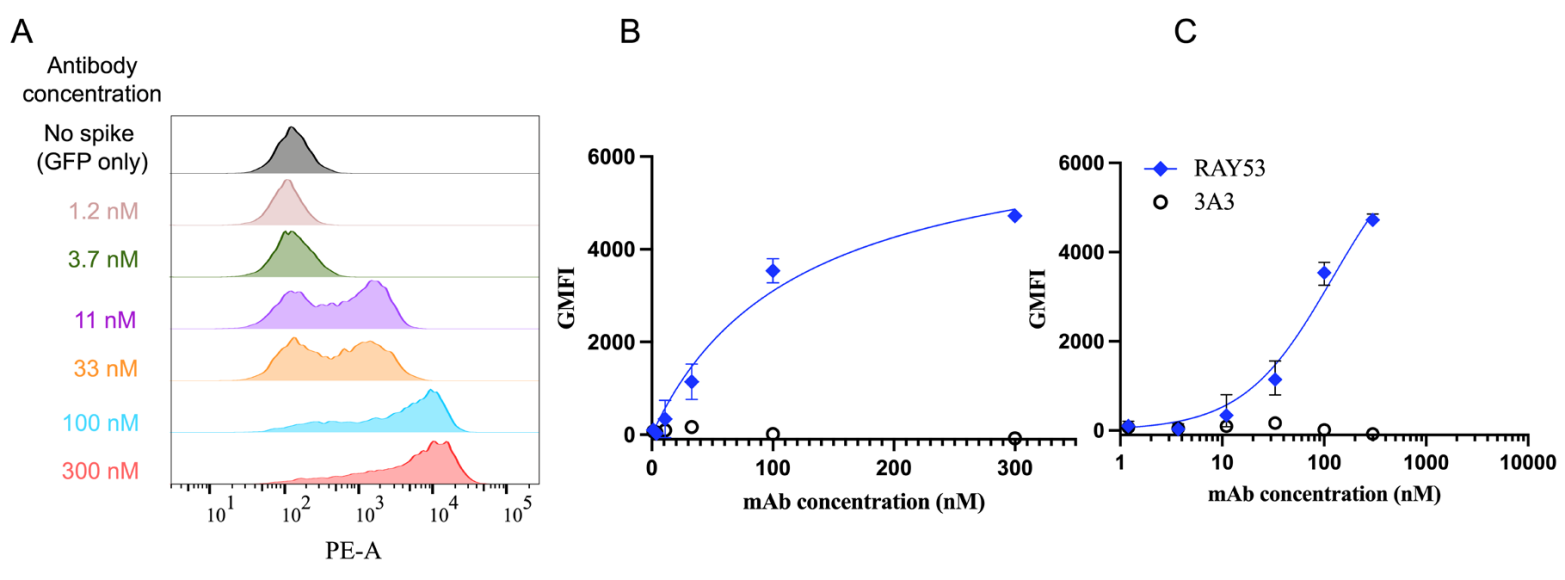
Determining effective K_D _from flow cytometry data. Data derived from the gating described in Figure 3 is transformed and plotted to determine data fits and effective K_D_ of anti-spike antibodies. A) After gating the cytometer data as shown in Figure 3, example histograms show a fluorescence shift (GMFI) corresponding to different RAY53 antibody concentrations against cells displaying Wuhan-Hu-1 spike. B) The population GMFI from the histograms in A is plotted against antibody concentration to form a Langmuir isotherm. C) Data can also be plotted log-transformed and used to determine effective K_D_ as described above. Plot taken from Silva et al. [24].

To determine the effective antibody K_D_, export the GMFI for each sample into Microsoft Excel or another spreadsheet software. For each antibody concentration, normalize each sample by subtracting the average GMFI of the pEGFP-only transfected wells stained with the same concentration of antibody. Upload the resulting normalized GMFIs with their corresponding antibody concentrations into GraphPad Prism or other plotting software. Visualize the Langmuir isotherm plot: antibody concentration vs. PE GMFI of EGFP positive cells, Equation 1. The plot should include the full curve, with multiple points in the initial increase phase as well as at least one point showing maximum binding. If there are one or fewer points in each phase, repeat the experiment with more points (Figure 4).

$$Equation\;1.\;C_{eq}=C_{max}\;\times \frac{L_{0}}{\;L_{0}+\;K_{D}\;}\;$$

Where C_eq_ is the concentration of complex (antibody bound to receptor, in this case sample GMFI), C_max_ is the maximum concentration of complex (here, the GMFI of our top concentration, ideally saturated), L_0_ is the concentration of ligand (here, the antibody), and K_D_ is the equilibrium binding affinity. A key assumption of the Langmuir analysis is that the antibody is always present in excess of the number of spike proteins and thus is constant regardless of the number of complex molecules formed; the testing outlined in the description of Part I, section B determines whether this assumption is met.

Next, create a semi-log plot with the log of antibody concentration on the x-axis and the PE GMFI of EGFP positive cells on the y-axis using GraphPad Prism. Under *Analysis*, click *Analyze* and then *Nonlinear regression (curve fit), Dose-response – Stimulation*, and choose *[Agonist] vs response (three parameters).* This program uses a slight variation of the Langmuir isotherm (**Equation 1**) that accounts for a non-zero minimum GMFI, listed below,

$$Equation\;2.\;Y=minimum+\left[\frac{\left(maximum-minimum\right)\;\times \;X}{{EC}_{50}+X}\right]$$

where minimum is the GMFI of the no-antibody control, maximum is the GMFI of the top concentration (C_max_), X is the concentration of antibody (L_0_), Y is the GMFI of the sample (C_eq_), and EC_50_ is the effective equilibrium K_D_. The calculated K_D_ will have the same units as the antibody concentration.

**Figure 5.**
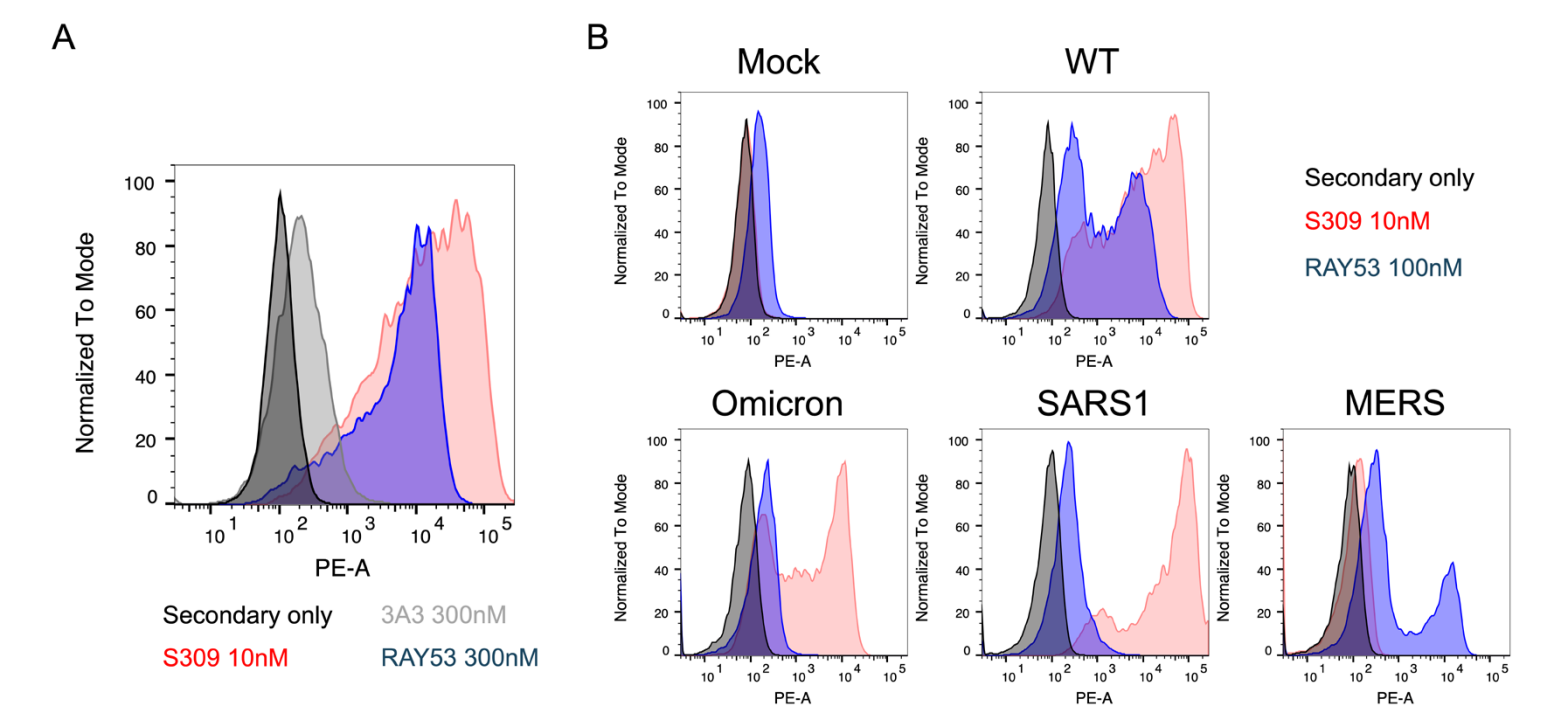
Antibody binding to cell surface–displayed spike variants. Example data comparing antibody binding to a panel of native coronavirus spike proteins. Each spike protein was expressed on the surface of Expi293F cells, stained with antibodies, and qualitatively characterized by flow cytometry. A) A histogram shows antibody binding to Wuhan-Hu-1 spike by the secondary-only negative control and S309 positive control and test antibodies 3A3 and RAY53. B) Binding of the 3A3 and RAY53 antibodies to cells displaying the indicated spike variants is compared by histograms of singlet cells. Differences in RAY53 binding and epitope accessibility can be evaluated by examining the loss of binding to spike with mutations in the epitope as described in Table 1.

To examine antibody cross-reactivity and epitope accessibility, two comparisons are made. First, if the epitope is known, the epitope amino acid sequence for each variant can be compared. Second, the binding histogram and GMFI should be compared to the positive control (S309) for each variant and compared between the variant and Wuhan-Hu-1 spike (Figure 5A). Ideally, choose a positive-control antibody whose epitope is available in most spike conformations to measure spike expression level. We also highly recommend choosing a control antibody well-characterized in the literature and known to bind various spike variants.

Before testing experimental samples, confirm the expression of each spike variant with the positive-control antibody. The respective binding (GMFI) of controls to variant spikes can be compared to known affinities in the literature. For example, S309 is known to bind SARS-1 and SARS-2 but not MERS [28]. Each experimental sample can then be compared to the positive control for each respective spike variant (Figure 5B). Large differences in transfection efficiency and display (≥10%) can impact comparisons across spikes and transfection should be optimized to minimize these differences if needed.

If there is no direct change in the sequence of the antibody epitope, changes in binding are most likely due to conformational differences in the spike variants. If there are identified mutations in the epitope, it may be difficult to confidently distinguish between changes in epitope affinity and epitope accessibility. Conclusions that may be drawn from various results are described below in Table 1.

**Table 1.** General guide to aid in determining the possible causes of antibody binding changes between spike variants.

Sequence comparison (variant vs. Wuhan-Hu-1)	Binding comparison (variant vs. Wuhan-Hu-1)	Cross-reactivity and epitope accessibility
No change	No change	Cross-reactive
No change	Significantly reduced, non-zero	Reduced epitope accessibility
No change	No binding	Severe or complete reduction in epitope accessibility
No change	Significantly improved	Cross-reactive, improved epitope accessibility
Mutation(s)	No change	Cross-reactive*
Mutation(s)	Significantly reduced, non-zero	Cross-reactive, potential loss of affinity*
Mutation(s)	No binding	Not cross-reactive*
Mutation(s)	Significantly improved	Cross-reactive*

*This analysis works best in cases where there is no direct change to the antibody epitope. In this case, observed changes in antibody binding can be attributed to differences in spike protein conformation and epitope accessibility. One caveat in evaluating cases with epitope changes is that an affinity loss can be offset by increased accessibility, or vice versa. This can make it difficult to discern whether changes are primarily due to affinity or epitope accessibility.

**Part II:**

In Microsoft Excel or another equivalent software, upload your fluorescence measurements from the plate reader. To calculate the percentage of target cells that were lysed through ADCC, use Equation 3 below:

Equation 3: 

$$Percent\;lysis=\left(experimental-spontaneous\;release\right)÷\left(max\;release-spontaneous\;release\right)\times \;100$$

Experimental: Sample of interest (i.e., target cells + NK cells + 10 nM antibody).

Spontaneous release: Sample containing target cells and NK cells but no antibody.

Max release: Sample containing target cells and NK cells with lysis buffer added (no antibody).

**Figure 6.**
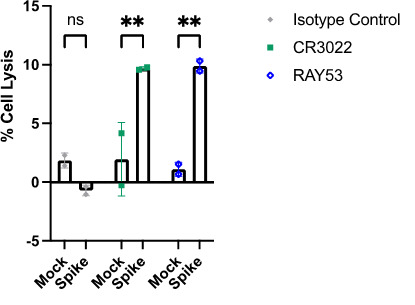
Analysis of antibody-dependent cellular cytotoxicity (ADCC) activity. ADCC activity (percentage target cell lysis) is compared between mock and spike-expressing target cells for an isotype antibody (grey), control antibody CR3022 (green), and RAY53 (blue). Two-way ANOVA was performed to compare samples with spike-displaying cells or mock-transfected cells for each antibody. ** denotes p < 0.1; ns, not significant. Figure from (Silva et al., 2023).

Values should be between 0% and 100%, although some non-effective samples will result in minor negative values. Values ranging beyond 0%–100% imply issues with non-specific activity in the negative control or low responses in the positive control, while large error among replicates can be due to high experimental variation among replicates (e.g., due to pipetting errors) or a small dose-response range, which is very sensitive to small differences. Additional factors that can contribute include evaporation causing edge effects, incomplete lysis of the control lysis wells, or cell clumps leading to inconsistencies among wells. The calculated percentage lysis values can be graphed as in **Figure 6**, to visualize comparisons and facilitate statistical comparisons.

## Validation of protocol

This protocol or parts of it has been used and validated in the following research article(s):

- Silva et al. [24]. Identification of a conserved S2 epitope present on spike proteins from all highly pathogenic coronaviruses. eLife. Effective antibody K_D_: Figure 4, panel e, ADCC: Figure 5, panel d.

Each point was performed with 2–3 technical duplicates and each experiment was repeated 2–3 times. Antibody binding controls included a single stain with the control antibody S309 at 10 nM (maximum staining, data not shown) to determine spike display level.

Validation of spike expression was performed using S309, also known as Sotrovimab, a well-characterized anti-spike antibody that is cross-reactive to SARS-CoV-2 variants and is known to have reduced binding to Omicron variants and no binding to MERS spike protein (Pinto et al. [28]).

## General notes and troubleshooting

**General notes**

1. Make sure to test the transfected cells with control antibodies to confirm that the spike is displayed. We recommend using S309. See Figure 3B for an example.
2. This protocol can be modified to test for antibody binding to various SARS-CoV-2 variant spike proteins by substituting the transfection plasmid DNA to include desired amino acid changes. The resulting antibody binding can be evaluated by a full dilution curve as above or by single antibody concentrations within the dose-response range to various spike variants. See Figure 5B for an example.
3. This protocol has been tested using other cell lines for spike expression, including 293F and ExpiCHO. While spike expression was detected, we also observed a higher percentage of dead cells than when using Expi293F. While dead cells can be gated out during analysis, they introduce potential sources of error, especially in cases of “sticky” antibodies.

**Troubleshooting**

**Part I: Effective K_D_ by flow cytometry**

Problem 1: No antibody staining observed.

Possible cause A: Antibody has a weak affinity.

Solution A: Increase the highest concentration of antibody used.

Possible cause B: Secondary antibody is inactive or incompatible with the primary antibody used.

Solution B: Check the compatibility of the primary antibody and the secondary antibody used in an ELISA or similar assay.

Problem 2: High percentage of dead cells.

Possible cause A: Contamination in DNA preparation, transfection reagents, or media.

Solution A: Filter-sterilize all reagents.

Possible cause B: Transfecting too much DNA.

Solution B: Repeat using lower DNA mass (or re-measure DNA concentration).

Possible cause C: Spike expression is toxic for the cells.

Solution C: Repeat using lower DNA mass.

Problem 3: High staining found in the negative controls (isotype antibody and/or secondary antibody–only controls).

Possible cause A: Non-specific binding by the isotype control or secondary antibody.

Solution A: Isotype and secondary antibodies can exhibit low non-specific binding to one cell line but be problematic for another. Accordingly, we first screen secondary antibodies, including those from different vendors and polyclonal vs. monoclonal options, anti-human-Fc, anti-human kappa, anti-human H+L, and F(ab)_2_ preparation of anti-human Fc to identify one with low non-specific binding to cells, similar to unstained cells. We then screen several candidate isotype control antibodies to find one that exhibits minimal non-specific binding. Extra washes and or different antibody concentrations may also be required.

Possible cause B: High percentage of dead cells.

Solution B: Dead cells will often bind to antibodies indiscriminately. Staining of untransfected cells with high viability can help determine if this is the issue. Follow the suggestions in Problem 2 to reduce the fraction of dead cells in your samples.

Problem 4: Staining by the experimental antibody is low or high across all concentrations tested.

Possible cause: Antibody concentrations used are outside the dose-response range.

Solution: If the signal is low, increase concentration. If the signal is high, decrease concentration. Perform a pilot experiment using a wide range of concentrations with 5-fold or 10-fold dilution steps to define those corresponding to maximum and minimum signals. Repeat the experiment using smaller dilution steps across the confirmed dose-response range.

**Part II: Antibody-dependent cellular cytotoxicity**

Problem 1: Final ADCC values above 100%.

Possible cause: Incomplete lysis in maximum lysis control or inconsistent cell numbers per well.

Solution: Make a new lysis buffer or optimize co-culture incubation time for complete lysis. Avoid clumps in initial 293T passaging, and pipette gently but thoroughly before and during cell seeding into plates.

Problem 2: Final ADCC values below 0%.

Possible cause: High spontaneous lysis or inconsistent cell numbers per well.

Solution: Make sure cells have good viability before seeding. To avoid edge effects, do not use the outer wells of the plate or fill the space between wells with PBS. Avoid clumps in initial 293T passaging, and pipette vigorously before seeding in plates.

Problem 3: High error between replicates.

Possible cause A: Inconsistent cell number per well or inconsistent calcein-AM loading due to clumps in target cells.

Solution A: Avoid clumps in initial 293T passaging, and pipette vigorously before seeding in plates. Additional parameters that may need to be optimized include E:T ratio, total number of cells, incubation times, and antibody concentration.

Possible cause B: Issues with the multi-channel pipette.

Solution B: Visually inspect multi-channel during pipetting to determine whether equal volumes are being administered by all channels.
